# A Prospective Study of a Custom-Made Eco-Friendly Thyroid Shield: A Breakthrough in Radiation Protection

**DOI:** 10.7759/cureus.75762

**Published:** 2024-12-15

**Authors:** Mulagolla Sirisha, Bharani Krishna Takkella, Jyothyrmayi Ambadipudi, Nakoor Akshath Rai, Rohit Vadlamani, Vallurupalli Jayanth, Kasireddy Naga Tejaswi, Chukka Ram Sunil, Sujanamulk Bhavana

**Affiliations:** 1 Department of Oral Medicine and Radiology, Konaseema Institute of Medical Sciences (KIMS) Dental College, Amalapuram, IND; 2 Department of Oral Medicine and Radiology, Drs Sudha and Nageswara Rao Siddhartha Institute of Dental Sciences, Vijayawada, IND; 3 Department of General Dentistry, Fresh Dental Smiles, Lauderhill, USA; 4 Department of General Dentistry, Jefferson Dental and Orthodontics, Dallas, USA; 5 School of Dentistry, University of Louisville, Louisville, USA; 6 Department of Medicine, Drs Sudha and Nageswara Rao Siddhartha Institute of Dental Sciences, Vijayawada, IND; 7 Department of Conservative Dentistry and Endodontics, Sibar Institute of Dental Sciences, Guntur, IND

**Keywords:** commercially available thyroid collar, custom made thyroid shield, dosimeter, equivalent dose, intra-oral radiography, radiation exposure, sv(sievert)

## Abstract

Background

The thyroid gland is the most susceptible organ to radiation during the exposure of teeth because the thyroid area appears to be within the primary beam, and the dose levels are relatively high even after using collimation. This study aims to develop an eco-friendly thyroid shield by reusing lead foils from intra-oral periapical radiographic films and evaluate its effectiveness in intraoral radiography.

Methods

A total of 16 patients undergoing endodontic procedures who gave written consent to participate in the study were included and divided into four categories: anterior, canine, premolar, and molar. After preparing a thyroid shield by reutilizing the lead foils, its effectiveness was checked and compared with that of a commercially available thyroid collar (CTC) by measuring the equivalent dose at the position of the thyroid gland while taking radiographs for all four categories. Instruments used included lead foils, cardboard, a CTC, a custom-made thyroid shield (CTS), a dosimeter, and X-ray film. Later, they were divided into three groups: Group I (without), Group II (with CTC), and Group III (with CTS).

Results

During the radiography, the CTS (Group III) reduced the radiation dose at the thyroid level by approximately 89% and was more effective than the CTC (Group II) in dose reduction when size 2 intraoral periapical (IOPA) radiographs were used.

Conclusion

The results of this study concluded that the CTS, besides its low cost and ease of preparation, is eco-friendly and more effective than the CTC in terms of dose reduction.

## Introduction

Oral health is an important determinant of overall health, as described by Jokovic A et al., Dion N et al., and Benyamini Y et al. [1-3]. Because patient dose remains a concern in diagnostic imaging, it is imperative that International Commission on Radiological Protection (ICRP) recommendations be investigated for commonly performed dental radiographic examinations, according to Davies LE et al. [4]. Mathur H et al. stated that with the advent of digital radiography, X-rays being produced were generally safer and more efficient, with 80-90% less radiation than traditional X-rays [5]. Many epidemiological studies have provided increasing evidence of thyroid tumors due to dental radiography, as advocated by Memon A et al. [6]. Kshyanaprava R et al. said that lead is one of the major harmful heavy metals in every ecosystem, leading to toxicity in humans as well as other vegetation. To make it favorable for the environment, already available lead must be renovated (reused and recycled), so that the toxic effects can be minimized [7]. The thyroid gland is one of the crucial organs of our body, which is accidentally exposed to primary or secondary (scattered radiation), as established by Tomohiro O et al. [8]. According to the Atomic Energy Research Board (AERB) guidelines, the thyroid shield is mandatory for all patients to avoid the risk of thyroid carcinoma, although many materials have been used for shielding crucial organs, and lead remains the prime metal; in recent times, lead-free collars have been produced and are widely used in dental imaging, as substantiated by Shannon F et al. [9]. In a study done by Kobayashi D et al. [10], using sodium iodide scintillation and Geiger-Muller survey meters to measure the radiation dose, it was proven that a wooden board or a magazine, either of them 20mm thick, is useful for radiation dose reduction. The percentage of radiation reduction would be much reduced for the thyroid gland upon the use of thyroid shields. In another study, Mohammad E et al. conducted thorough research using newly developed polymers and found a direct relation between the use of these polymers, like high concentration WO3, and profound radiation dose reduction [11]. Even though commercially available thyroid collars (CTC) have been used for a long time, an attempt at customized thyroid shield fabrication has not appeared much in the literature. Therefore, the current study aimed at the fabrication of a custom-made thyroid shield (CTS) by reusing the lead foils from conventional intra-oral radiographs while exposing different segments in the oral cavity.

## Materials and methods

Study commencement date

The study procedure began on November 12, 2023, and continued until September 30, 2024, at Drs. Sudha and Nageswararao Siddhartha Institute of Dental Sciences, Chinnaoutpalli, Andhra Pradesh, India. The institute's ethical committee number was O.C. No./IEC/31/2023. All patients were thoroughly explained about the study and written consent was obtained from those who were willing to participate.

Materials used for the preparation of the CTS

Materials such as lead foil, customized cardboard, scissors, glue, a handle, an impervious cover, and plaster were used to fabricate the CTS.

Materials used to evaluate the efficacy of CTC and CTS

The X-ray machine (Blue X with 70 kVp, 7 mA) manufactured by KONA Srl Assago, Italy, was used, along with a commercially available pocket dosimeter (UNI-T-334 A Weiger counter) and intraoral periapical (IOPA; Size 2 measuring 31 mm x 41 mm), CTC, and CTS. The radiation entity measured was the equivalent dose at the level of the thyroid gland, measured in sieverts (Sv).

Study design and procedures

Preparation of CTS

**Figure 1:**
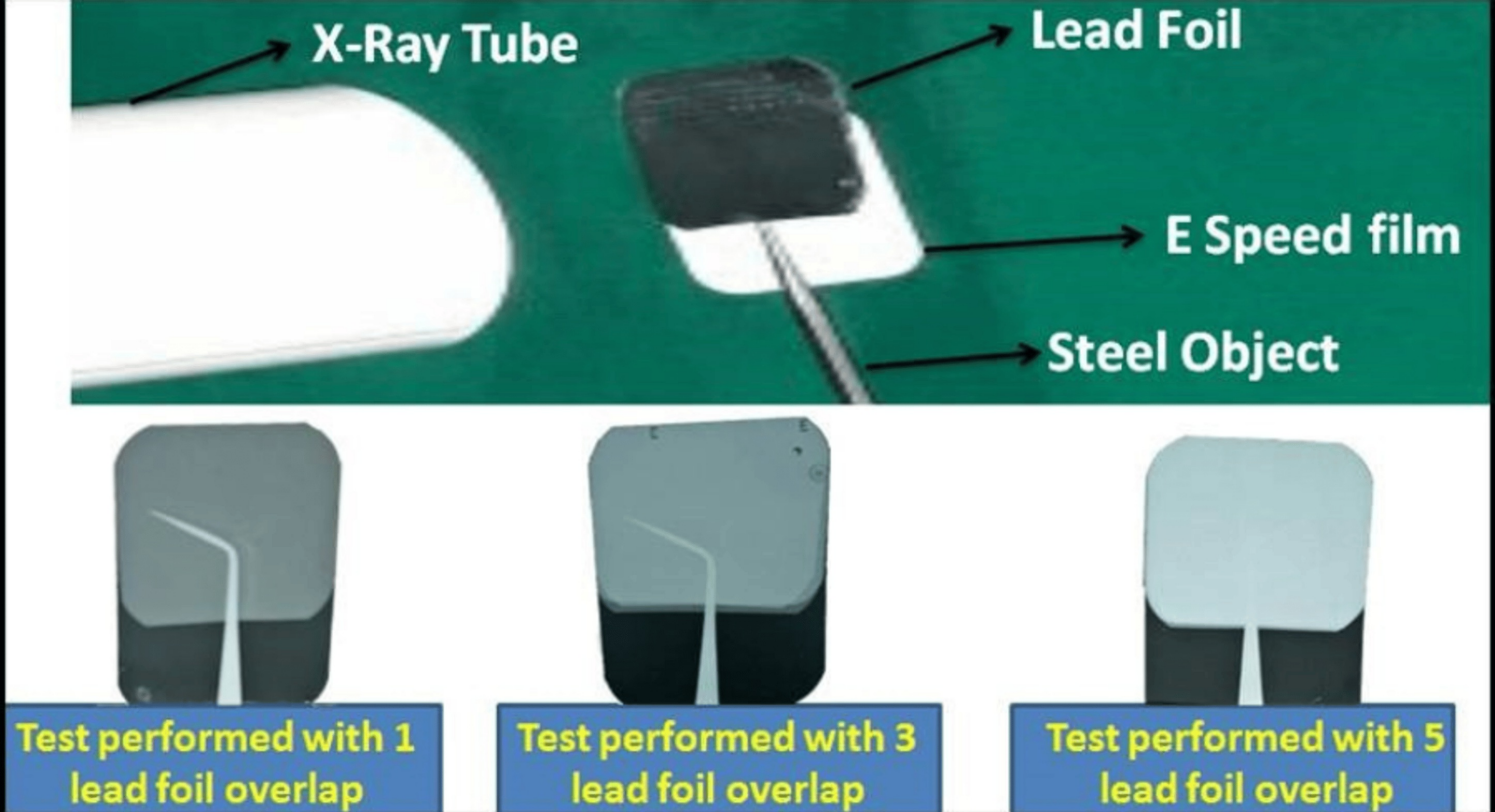
Preparation of custom-made thyroid shield.

For preparing the CTS, lead foils used in the preparation were carefully isolated from the radiographic films and disinfected with a glutaraldehyde solution activated by 2% sodium bicarbonate. These were later transferred to a clean and dry working room equipped with good illumination, where they were customized with other materials. To determine the thickness of the thyroid shield, a small test was performed, where an object of steel was placed between the lead foil and the periapical film, which was then exposed to the dental IOPA machine. This test was repeated until an invisible image was obtained by increasing the number of lead foils (Figure 1).

**Figure 2:**
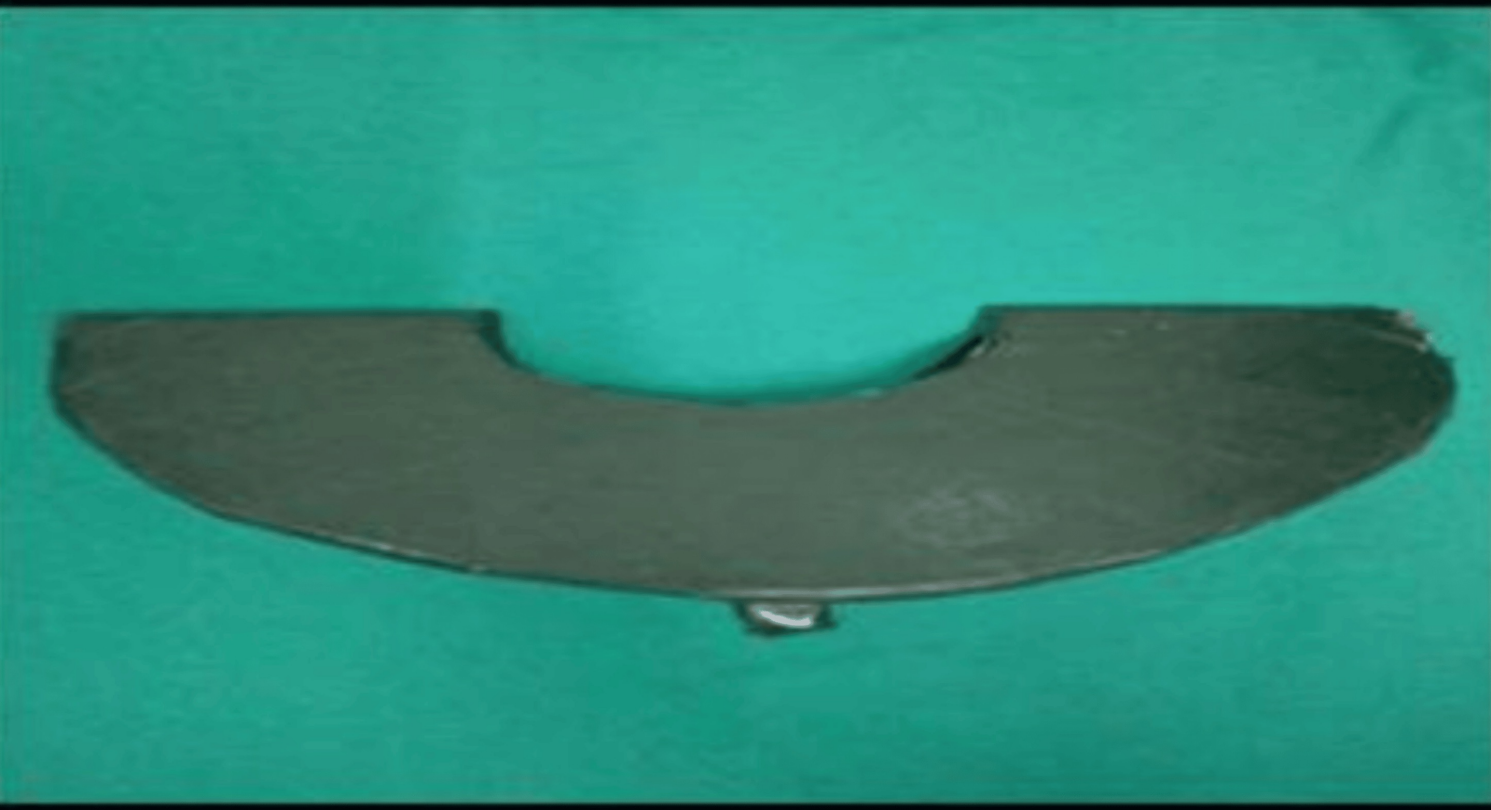
Custom-made thyroid shield.

Finally, when five lead foils were put together, we could achieve the desired results, i.e., the image portion of the object under the lead foil was not visible. For better results and to avoid radiation leakage, we made a thyroid shield with the thickness of seven lead foils, which were arranged in an overlapping manner by sticking them to each other and spreading over a 'C'-shaped cardboard; the whole assembly was then covered with an impervious cover and equipped with a handle (Figure 2). The thickness of the lead foils measured during the fabrication of the thyroid shield was found to be 0.54 mm, which is in accordance with AERB guidelines for radiation safety and protection [9].

Procedure for Evaluation of CTS Efficacy

**Figure 3:**
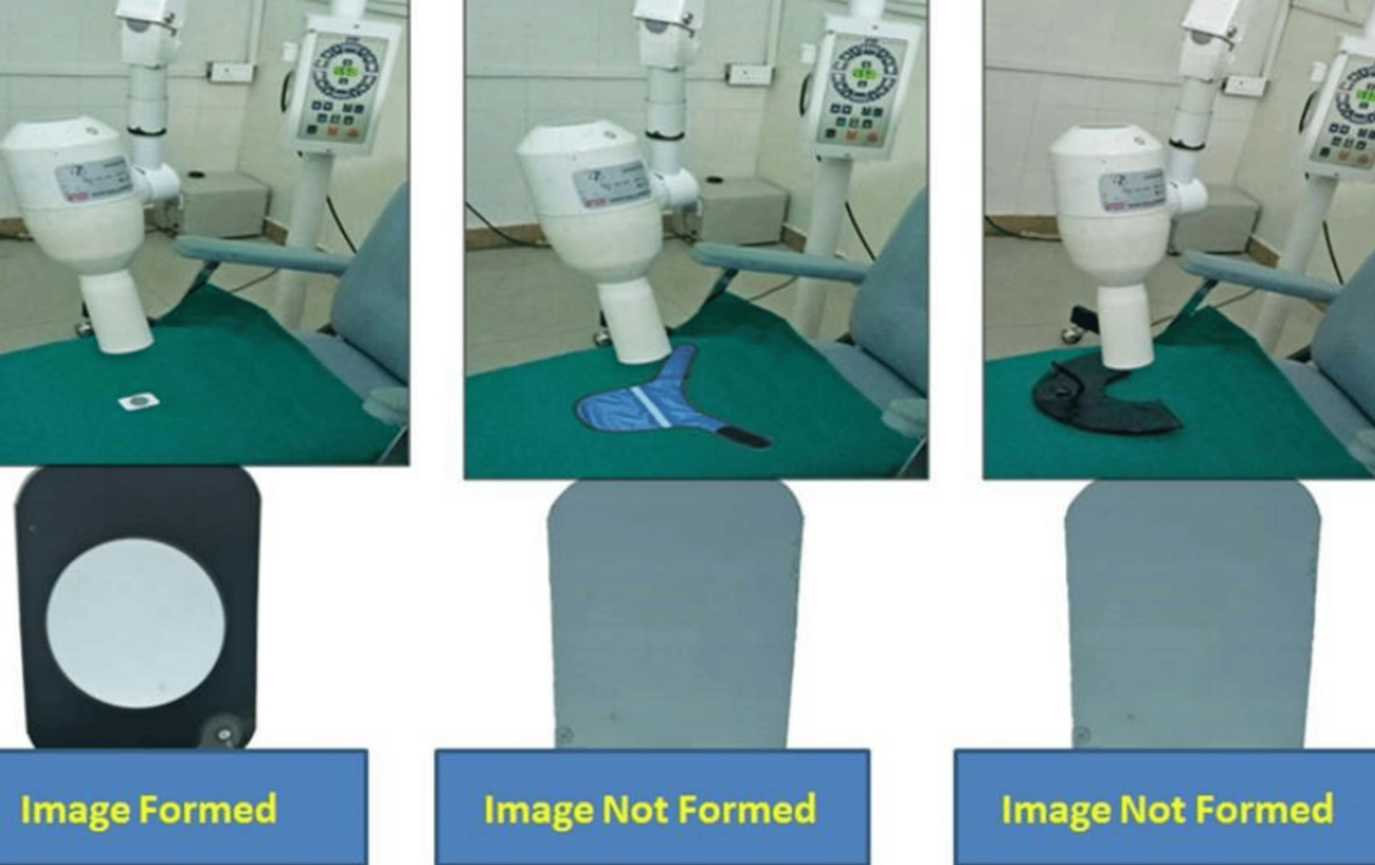
Procedure for evaluating custom-made thyroid shield efficacy.

Before using it clinically, the efficacy of the CTS was checked by a simple coin test. A coin was placed over a film and exposed, and then the image of the coin was formed. The same test was repeated with both CTS and the CTC over the coin. In both cases, the image of the coin was not formed, which indicates that both CTC and CTS were efficient in obstructing the X-rays (Figure 3).

**Figure 4:**
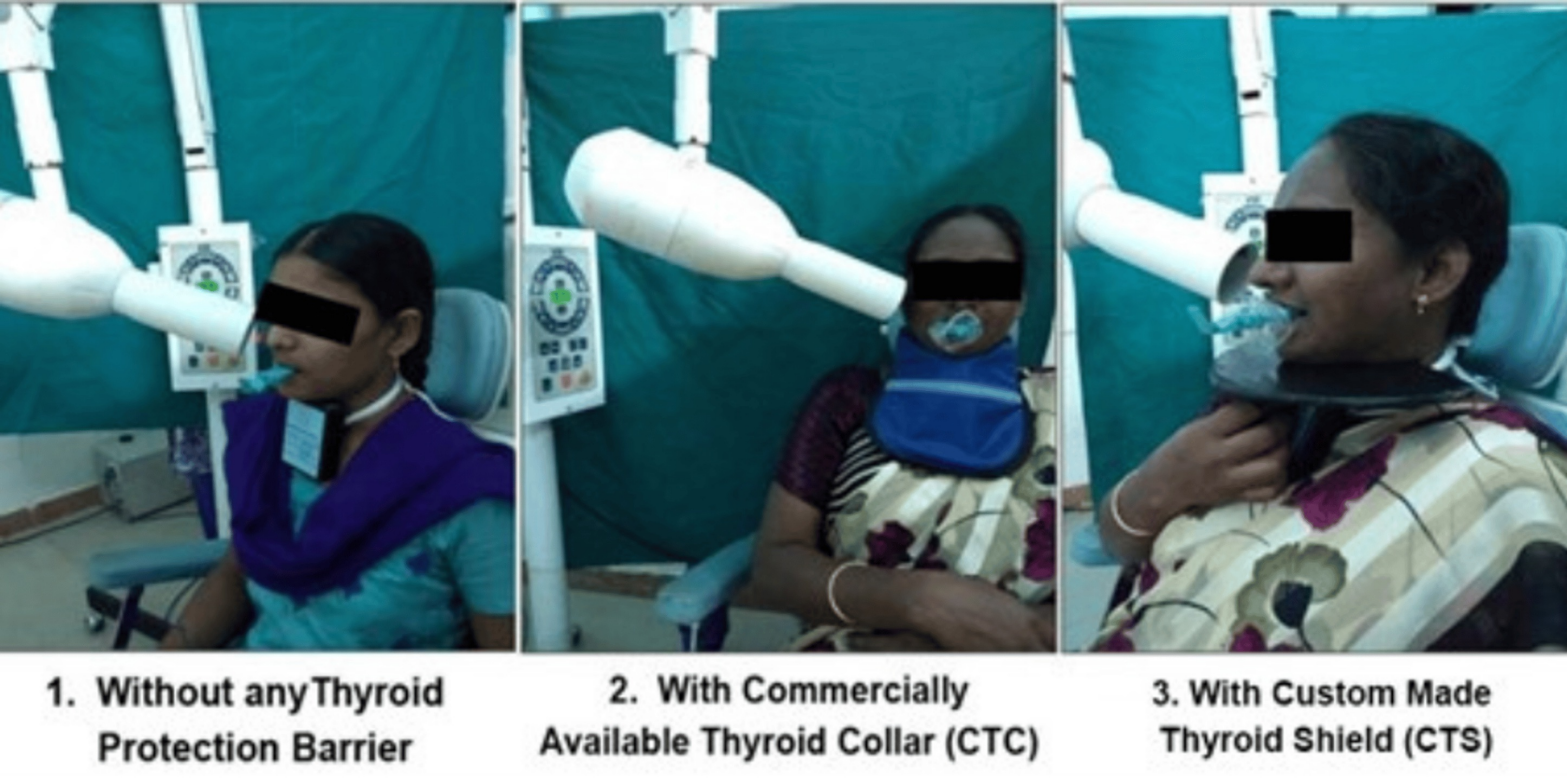
(1) Evaluation of a patient without any thyroid protection, (2) With a commercially available thyroid collar, and (3) With a custom-made thyroid shield.

Therefore, we used it clinically where a total of 16 patients undergoing root canal therapy for maxillary teeth were included. Based on the area of their requirement, they were divided into four categories - anterior (both central and lateral incisors), canine, premolar, and molar, with four patients under each category. For every patient, a pocket dosimeter of PD716 was tied to the lower part of the user’s neck covering the thyroid gland. The exposures were performed during endodontic treatment (preoperative, working length, and post-obturation) in three different groups, i.e., without any (Group I), with CTC (Group II), and with CTS (Group III) under the same exposure parameters and also ensuring that there was no change in the position of the patient’s head during the procedure. Since the lead apron emits a small amount of stray radiation, it was intentionally avoided to prevent errors while recording the efficacy of CTS and CTC, which was clarified to all the patients participating in the study and also their consent was obtained (Figure 4).

Statistical analysis

The statistical analysis was performed using IBM SPSS ver. 22 (IBM Corp., Armonk, NY, USA). Both descriptive and analytical data were determined. The normality of data was analyzed using the Shapiro-Wilk test. As the data followed a normal distribution, parametric tests were used to analyze the data. The ANOVA test with Tukey's HSD post hoc analysis was used to check differences in mean scores among the groups. Statistical significance was set at p<0.05, which was considered a significant association at the 5% level of significance, whereas p<0.001 was considered highly significant, and p>0.05 was said to be non-significant.

## Results

The study demonstrating the exposures along with the vertical angulation for various teeth has been described in Table 1. The numerical values of the equivalent dose exposed (SI unit is sievert), measured in microns microsievert (µSv), are depicted in Table 2.

**Table 1:** Exposure and angulation settings for different geometries of the selected teeth.

Tooth	Vertical angulation	Exposure time (msec)
Anterior	40°	350
Canine	45°	490
Premolar	30°	550
Molar	20°	650

**Table 2:** Table representing the numerical values of radiation parameters for each group: GI - without thyroid shield, GII - commercially available thyroid collar (CTC), and GIII - custom-made thyroid shield (CTS).

Tooth	GI (µSv)	GII (µSv)	GIII (µSv)
Anterior			
1	12.74	2.29	1.05
2	11.26	2.09	1.013
3	12.04	2.19	1.04
4	14.09	2.32	1.07
Canine			
1	9.45	2.07	1.04
2	9.02	2.07	1.03
3	8.96	2.01	0.98
4	9.56	2.12	1.07
Premolar			
1	2.09	1.46	1.04
2	2.07	1.43	1.04
3	2.06	1.47	1.07
4	2.04	1.41	0.99
Molar			
1	2.02	1.43	0.96
2	2.01	1.24	1.02
3	1.98	1.36	1.07
4	2.08	1.02	0.97

Mean doses at the site of the thyroid gland for four different geometries with three different modalities are shown. When Groups I and II were compared for all the patients among the four modalities (anterior, canine, premolar, and molar), there was a statistically significant reduction in radiation with (p<0.001), indicating that the CTC can shield radiation efficiently. Similarly, when Groups I and III were compared across all teeth, the radiation reduction was found to have clear-cut statistical significance with (p<0.001), indicating the potency of the CTS in radiation protection. When Groups II and III were compared, there was no statistically significant reduction for anterior teeth, whose (p=0.093); however, for canine and premolar teeth, the reduction was highly statistically significant with (p<0.001), but for molar teeth, there was statistical significance with (p=0.021) (as we considered p<0.05 as a significant association at the 5% level of significance), as depicted in Table 3.

**Table 3:** Comparison of mean radiation doses at the site of the thyroid gland for four different tooth regions among three groups - Group I: without a thyroid shield, Group II: commercially available thyroid collar (CTC), and Group III: custom-made thyroid shield (CTS). CTC: Commercially available thyroid collar; CTS: Custom-made thyroid shield.

Number of teeth	Tooth	Mean ± SD				Multiple comparisons of the groups			
		GI - W/O	GII - CTC	GIII - CTS	GI vs GII			GI vs GIII	GII vs GIII
4	Anterior	12.53± 1.20	2.22± 0.10	1.04±0.02	<0.001			<0.001	0.093
4	Canine	9.24± 0.30	2.06± 0.04	1.03± 0.03	<0.001			<0.001	<0.001
4	Premolar	2.06± 0.02	1.44± 0.02	1.03± 0.03	<0.001			<0.001	<0.001
4	Molar	2.01± 0.02	1.26± 0.17	1.00± 0.05	<0.001			<0.001	0.021

When the custom-made thyroid shield was used, there was a greater decrease in radiation for molars, followed by its use with canines, anteriors, and premolars, compared to that of the commercial thyroid collar. During intraoral periapical radiographic exposures of all the teeth in the three groups, statistical data revealed mean values with a 95% confidence interval (CI) in radiation reduction among all three groups. When anterior teeth were examined using CTC, the dose reduction was found to be 82.20%, with upper and lower boundaries of 81.58-83.10 at 95% CI. Similarly, for CTS during the examination of anterior teeth, the dose reduction was 91.13%, with lower and upper limits of 91.19-92.14 at 95% CI. Likewise, during the examination of canines using CTC at 95% CI, the lower and upper limits were 77.24-77.96, and the reduction rate was determined to be 77.63%. The reduction rate for canines with CTS was 88.86%, with lower and upper limits of 88.68-89.02 at 95% CI. During the evaluation of premolars using CTC, the reduction rate was 30.14%, whose lower and upper boundaries were 29.20-30.90 at 95% CI. Using CTS, the reduction rate was 37.14%, with lower and upper limits of 30.26-45.14 at 95% CI. When molars were evaluated using CTC at 95% CI, with lower and upper boundaries known to be 30.26-45.14, the reduction rate was 37.14%. By using CTS for molars, the reduction rate is 49.97%, with upper and lower limits of 47.52-52.34 at 95% CI, as depicted in Table 4.

**Table 4:** Radiation reduction at the site of the thyroid gland for four different tooth modalities using a commercially available thyroid collar (CTC) and a custom-made thyroid shield (CTS).

Tooth	N	Group	Reduction (%)	95% CI
Anterior	4	CTC	82.2	81.58-83.10
		CTS	91.13	91.19-92.14
Canine	4	CTC	77.63	77.24-77.96
		CTS	88.86	88.68-89.02
Premolar	4	CTC	30.14	29.20-30.90
		CTS	37.14	30.26-45.14
Molar	4	CTC	37.14	30.26-45.14
		CTS	49.97	47.52-52.34

## Discussion

With the discovery of x-rays on November 8, 1895, by German physicist Wilhelm Conrad Röntgen, the golden era of radiology dawned. X-rays, a type of electromagnetic radiation, are used in most imaging modalities, as stated by Panchbhai AS et al. They have both advantages and disadvantages. Advantages include the identification of cracks, infections, injuries, abnormal bones, bone cancer, locating objects inside and around bones, and treating malignant tumors before metastasis. Due to these incalculable benefits, their usage has increased remarkably, although the disadvantages may outweigh the advantages of X-rays [12]. And, as stated by Linn MC et al., disadvantages include cell damage by increasing hydrogen peroxide levels in a cell and an increased risk of mutations at the base of DNA leading to cancers [13]. In a study on a phantom to evaluate the utility of a thyroid shield in intra-oral radiography done by Hoogeveen RC et al., it was concluded that using a thyroid shield during exposure of the maxillary central incisors resulted in a reduction of radiation dosage up to 74.78% [14]. Current study findings reveal that radiation dosage to the thyroid during exposure of the maxillary central incisors reduced by 91.63% when a custom-made thyroid shield was used, and 82.20% with a thyroid collar. In another study done by Alkhateeb SM et al. to evaluate radiation safety for the thyroid gland in chest X-rays, they found that a reduction rate of 69% ± 18% was achieved by using a thyroid shield [15]. Likewise, in our study, when maxillary canines were exposed, a dose reduction of 88.86% and 77.63% for the thyroid gland was seen when CTS and CTC were used, respectively.

Hende WR advocated that the International Council for Radiation Protection (ICRP) in its 1990 recommendations introduced the concept of optimization - the principle of as low as reasonably achievable (ALARA) [16]. There is a saying, 'Anything over in use is harmful.' ALARA is a safety principle designed to minimize unnecessary exposures as well as overexposure, with social and economic factors being taken into account. According to Sodhi KS et al., the optimization concept has been altered from the beginning as a result of increasing knowledge of radiation and its effects on people. X-rays, which have innumerable benefits, cannot be avoided, but the adverse effects can be minimized by taking a few precautions [17]. Berkhout WE et al. proved that one of the best precautionary measures to reduce the radiation dose is by shielding [18]. Lead is an excellent material of choice for shielding, as documented by Hashimi SA et al. and McCafferey JP et al. [19,20], which coincides with the current study where lead is the source material used in the preparation of CTS.

Commercial lead is of two types, primary and secondary. According to Shukkla V et al., the source for primary lead is a mixed lead core, while secondary lead is extracted from the scrap of lead products, which are recycled by a process called smelting [21]. In the current study, the lead used for manufacturing the thyroid shield is sourced from secondary lead. Three million tons of lead are produced from secondary sources every year by recycling [21]. In a study done by Wang L et al., they depicted that the smelting process releases a number of hazardous fumes and specks of dust into the environment. The lead particles, which are less than 10µ, are absorbed into the body primarily through inhalation, secondarily through ingestion, and to a minimal extent transdermally, leading to several unwanted effects like disruption of the biosynthesis of hemoglobin leading to anemia, hypertension, renal damage, miscarriages and abortions, disruption of the nervous system, brain damage, loss of libido, decreased learning ability, and impulsive behavior [22]. Despite numerous health concerns, lead is extensively used for the construction of shield barriers as it has a high atomic number, greater density, high level of stability, ease of fabrication, a high degree of flexibility, availability, economical value, reusability, and uniform density [21-22].

The dosage varies greatly for each approach in dental radiography. According to Ludlow JB et al., compared to intraoral radiographic procedures, extraoral radiographic techniques expose the patient to higher radiation doses. The relationship between radiation effects and equivalent dose is direct [23]. The first case-control investigation on the relationship between laryngeal cancer and dental X-rays was carried out by Hinds MW et al., but due to the small sample size, no conclusions could be drawn [24]. In a follow-up investigation about cellular alterations after dental X-ray exposure, Cerqueira EM et al. concluded that significant apoptotic nuclear variations suggestive of apoptosis were seen in the mucous membrane cells and periodontal cells [25]. Likewise, in another study done by Hohl C et al. to test the efficacy of thyroid and breast shields made of bismuth for radiation dose reduction in multi-detector computed radiography (MDCT) using phantom models, it was determined that using a thyroid shield reduced radiation by 47% and breast by 32% [26]. In our study, by using a lead shield, we observed a dose reduction of 37.14% and 30.14% for custom-made thyroid shields and commercially available thyroid collars, respectively, when maxillary premolars were exposed to diagnostic radiation.

According to a prospective cohort study by Han MA et al., there was a 13% increase in the incidence of thyroid malignancies for every ten documented dental radiographs. They reported that more than 62,000 new cases of thyroid cancer, with a high mortality rate, occurred in the United States in 2015. Childhood leukemia patients who received prophylactic cranial irradiation have a multifold increase in the risk of thyroid malignancies. There is also a substantial risk of developing hypothyroidism due to lack of safe exposure as even minute radiation is dangerous, owing to its cumulative effects over a lifetime [27]. In a study by Moghadam MS et al. [28] to compare the efficacy of lead and non-lead thyroid shields in reducing radiation, it was concluded that non-lead thyroid shields could reduce the radiation dose to 39.79%, and lead shields can nullify up to 21.49%. Han GS et al. [29] mentioned that by using thyroid protection during orthopantomographs, the amount of radiation dose for the thyroid gland was considerably reduced to 33%. In our study, it was found that when maxillary molars were exposed to dental X-rays, using CTS and CTC can shield the radiation to levels of 49.17% and 37.14%, respectively.

According to Wani AL et al., conventional radiographs must be managed properly by the aid of proper handling of lead foils, avoiding direct or indirect contamination with food and water; they must also be stored, used, and disposed of properly wherever they are utilized to prevent the risk of potent lead toxicity [30]. In this study, we created a thyroid shield simply by taping lead foils together where they were handled effectively following all necessary requirements and also without subjecting them to smelting, which helped to counteract the negative effects of lead toxicity. The CTS is highly cost-effective, convenient, and user-friendly both to the patient and to the dentist. The custom-made thyroid shield, besides its low cost, provided an opportunity to reuse the lead foils that can contribute significantly to the sustenance of the environment and also in terms of dose reduction, which is more effective than the commercially available thyroid collar. To reduce patients' exposure and ease patients' concerns about dental radiography, all practitioners should follow appropriate safety precautions.

Limitations

The sample size selected was small; better results can be expected if the sample size were larger. Considering IOPA radiograph, which was analog, now being replaced by digital, i.e., radiovisiography (RVG), in all parts of the world is a drawback of this study.

Future scope

The current study ensures that reusable lead from any source can act as a protective barrier from harmful radiation and thus prevent the buildup of lead in the environment and promote the use of inexpensive protective barriers over marketable radiation shields. Hence, saving ecology and economy. However, further longitudinal studies must be done to evaluate the efficacy and safety of CTS in extraoral radiographic procedures, which play a pivotal role in oral and maxillofacial radiology.

## Conclusions

In the current study, it was demonstrated that the CTS is more effective than the CTC in terms of dose reduction. The advantages of CTS include its low cost and the reusability of lead foil, which can significantly contribute to environmental safety. Therefore, in the near future, to reduce patient exposure and alleviate concerns about dental radiography, all practitioners could adopt CTS in their daily dental practice, following appropriate precautions with minimal requirements and cost-effective measures.
